# Separation of metabolic supply and demand: aerobic glycolysis as a normal physiological response to fluctuating energetic demands in the membrane

**DOI:** 10.1186/2049-3002-2-7

**Published:** 2014-06-05

**Authors:** Tamir Epstein, Liping Xu, Robert J Gillies, Robert A Gatenby

**Affiliations:** 1 Program of Cancer Biology and Evolution, Moffitt Cancer Center, 12902 Magnolia Drive, Tampa, FL 33612, USA; 2 Department of Cancer Imaging and Metabolism, Moffitt Cancer Center, 12902 Magnolia Drive, Tampa, FL 33612, USA; 3 Department of Radiology and Program of Cancer Biology and Evolution, Moffitt Cancer Center, 12902 Magnolia Drive, Tampa, FL 33612, USA

**Keywords:** Glucose metabolism, Warburg effect, aerobic glycolysis, membrane activity

## Abstract

**Background:**

Cancer cells, and a variety of normal cells, exhibit aerobic glycolysis, high rates of glucose fermentation in the presence of normal oxygen concentrations, also known as the Warburg effect. This metabolism is considered abnormal because it violates the standard model of cellular energy production that assumes glucose metabolism is predominantly governed by oxygen concentrations and, therefore, fermentative glycolysis is an emergency back-up for periods of hypoxia. Though several hypotheses have been proposed for the origin of aerobic glycolysis, its biological basis in cancer and normal cells is still not well understood.

**Results:**

We examined changes in glucose metabolism following perturbations in membrane activity in different normal and tumor cell lines and found that inhibition or activation of pumps on the cell membrane led to reduction or increase in glycolysis, respectively, while oxidative phosphorylation remained unchanged. Computational simulations demonstrated that these findings are consistent with a new model of normal physiological cellular metabolism in which efficient mitochondrial oxidative phosphorylation supplies chronic energy demand primarily for macromolecule synthesis and glycolysis is necessary to supply rapid energy demands primarily to support membrane pumps. A specific model prediction was that the spatial distribution of ATP-producing enzymes in the glycolytic pathway must be primarily localized adjacent to the cell membrane, while mitochondria should be predominantly peri-nuclear. The predictions were confirmed experimentally.

**Conclusions:**

Our results show that glycolytic metabolism serves a critical physiological function under normoxic conditions by responding to rapid energetic demand, mainly from membrane transport activities, even in the presence of oxygen. This supports a new model for glucose metabolism in which glycolysis and oxidative phosphorylation supply different types of energy demand. Cells use efficient but slow-responding aerobic metabolism to meet baseline, steady energy demand and glycolytic metabolism, which is inefficient but can rapidly increase adenosine triphosphate (ATP) production, to meet short-timescale energy demands, mainly from membrane transport activities. In this model, the origin of the Warburg effect in cancer cells and aerobic glycolysis in general represents a normal physiological function due to enhanced energy demand for membrane transporters activity required for cell division, growth, and migration.

## Background

In 1867 Pasteur demonstrated that yeast decrease ethanol production following aeration of the culture media [1]. This observation has led to an enduring paradigm that in the absence of pathology, glucose metabolism is predominantly governed by oxygen concentrations. Thus, high-efficiency oxidative phosphorylation (up to 36 ATP/glucose) is generally assumed to be the default source of ATP under normoxic conditions, whereas in the Emden-Meyerhoff fermentative pathway, glycolysis (glucose metabolized to lactate yielding 2 ATP/glucose), is an emergency back-up to be used when oxygen is deficient [2]. Observed glucose metabolism in most mammalian cells is consistent with this Pasteur effect, although some lactate production is typically observed even in the presence of oxygen [3-8]. Cancer cells, on the other hand, typically exhibit high rates of glucose fermentation in the presence of normal oxygen concentrations (aerobic glycolysis). This phenomenon, also termed the Warburg effect [9], has been recognized for nearly a century.

Aerobic glycolysis is widely viewed as a ‘deregulation of cellular energetics’ and thus, a hallmark [10] of cancer. However, decades of research to uncover the molecular source of abnormal cancer metabolism have failed to identify a consistent etiology. It was originally ascribed to a defect of oxidative metabolism [11], but mitochondrial dysfunction is observed in only a small subset of cancers [12,13]. Alternatively, it has been suggested that the Warburg effect, through its production of lactate, provides necessary carbon substrate for biosynthesis of macromolecules [14,15]. However, experimental observations [16] have demonstrated that only a very small percentage of glucose carbons (<7%) is retained in the cancer cell, while glutamine serves as the major source for anabolic carbon and nitrogen.

Failure to identify a consistent pathological basis for aerobic glycolysis [17], even after several decades of investigation, led us to question the basic assumption that glucose metabolism is governed solely by oxygen concentrations, that is, aerobic glycolysis represents disordered cellular energetics only in the context of the standard paradigm of oxygen-controlled ATP production. If one accepts the assumption that glycolysis, because of its inefficiency in ATP production, is used only when oxygen is inadequate then glycolysis in normoxic conditions must be disordered. Here we provide experimental and computational evidence to support an alternative demand-driven model in which glycolysis and oxidative phosphorylation are complementary production modes that supply ATP to different cellular processes with different timescales of energy demands. In our model, cells exploit the efficiency of aerobic metabolism to meet baseline, steady-energy demand, and the rapid response time of glycolysis is used to meet short-timescale energy demands, mainly from membrane transport activities. This model of glucose metabolism resembles the economic model of power-grid optimization. As in power grids, the activity of each metabolic pathway is governed by the magnitude of the energy demand that it meets. For instance, the rate of glycolytic metabolism is primarily determined by the activity of membrane transport. As such, a chronic increase of glycolytic metabolism, such as the Warburg effect in cancer, may be a physiological response to increased energetic demand due to enhanced membrane transport activity required to cell division, growth, and migration [18-21].

## Methods

### Cell culture

Cultures were maintained in standard incubation conditions, 37°C and 5% CO_2_ culturing media as follows: HMEC (Invitrogen Life Technologies Corporation, Carlsbad, CA, USA) and HuMEC Basal Serum Free Medium (Invitrogen) supplemented with HuMEC Supplement and Bovine Pituitary Extract (Invitrogen); MCF10a (ATCC, Manassas, VA, USA) and Dulbecco's modified Eagle's medium (DMEM)-F12 (Invitrogen) and 5% HS (HyClone Laboratories, UT, US); MCF7 (ATCC) and RPMI1640 (Invitrogen) + 10% FBS (HyClone Laboratories); MDA-MB-231 (ATCC, 2007–2010), DMEM-F12 (Invitrogen) and 10% FBS (HyClone Laboratories); MCF7/DOX: detailed description of culturing and cloning procedures can be found in a previous publication [22].

### Immunofluorescence staining

HMEC cultured on collagen-coated 1.5 coverslips for 24 hours were washed with 3X PBS, fixed in 4% paraformaldehyde for 10 minutes, permeabilized in 0.1% Triton for 10 minutes, and blocked in 1% BSA/1X PBS for 30 minutes. After washing cells in 3X PBS, they were serially incubated in Anti-Pyruvate Kinase antibody (1:500, Abcam, MA, US, ab6191), Goat antibodies were conjugated to a secondary Alexa 488 antibody 1:500. The coverslips were then mounted on microscope slides using Vectashild (Vector Laboratories, Burlingame, CA, USA) containing 4',6-diamidino-2-phenylindole (DAPI) for nuclei staining. Images were captured with a Leica TCS SP5 (Leica Microsystems Germany) confocal microscope.

### Mitochondrial staining

HMEC cells were seeded in 35-mm glass bottom plates (50 K cells/mL). They were incubated at 37°C/5% CO_2_ for 24 hours and then incubated with 0.5 mg/mL Mito Tracker Green for 15 minutes and washed with 1X PBS. Media were replaced prior to imaging.

### Microscopy

Micrographs of HMEC cells in glass-bottom plates or coverslips were taken with a Leica TCS SP5 AOBS laser scanning confocal microscope through a 63X/1.40NA Plan Apochromat oil immersion objective lens (Leica Microsystems). A 405-nm diode laser line was applied to excite DAPI nuclear dyes, an Argon (Lasos, Germany) 488-nm laser was used to excite Mito Tracker Green and Alexa 488 antibodies.

### Metabolic profiling of membrane activity

Metabolic profiling was conducted using the Seahorse XF reader (Seahorse Bioscience, Chicopee, MA, USA). Cells were seeded in XF 96-well cell culture microplates (Seahorse) at 12.5 × 10^3^ cells/well (0.32 cm^2^) in 80 μL growth medium, left at room temperature for 45 minutes and then incubated at 37°C/5% CO_2_ for 20 to 24 hours. Assays were initiated by removing the growth medium from each well and replacing it with 180 μL basic non-buffered media (see below), supplemented with glutamine 2 mM glutamine, 0.5 mM pyruvate and 17.5 mM glucose, washed three times by replacing 100 μL of the media and then were incubated at 37°C with no CO_2_ for 45 minutes, to equilibrate. After calibration and equilibration measurements of oxygen consumption rate (OCR) and proton production rate (PPR) were taken simultaneously in intervals of 6 minutes that included 2 minutes mixing and 4 minutes of measurements. The values of OCR and PPR reflect the activities of aerobic respiration and glycolysis respectively. The experimental protocol consisted of six basal measurements, followed by the reagent injection at different concentrations (six wells for every concentration) and additional hours of measurements, except for Verapamil where the post-injection measurements were 2 hours.

Ouabain and Verapamil (Sigma-Aldrich, O3125 and 1711202, MO, US) were dissolved in the non-buffered media. Gramicidin A (Sigma-Aldrich, 50845) was first dissolved in DMSO (7.52 mg in 100 μL) and then added to a non-buffered emulsion supplemented with Antifoam B (1:100 v/v, Sigma-Aldrich, A5757) to improve solubility.

Seahorse non-buffered DMEM media were as follows: 8.3 g/L Standard DMEM Powder (Sigma-Aldrich); 1.85 g/L NaCl; and 15 mg/L Phenol Red. Change in PPR and OCR were calculated as:

$$\frac{\text{Rate}\left(t_{0}+t'\right)}{\text{Rate}\left(t_{0}\right)}/\frac{\text{Rat}{e_{c=}}_{0}\left(t_{0}{+}^{t'}\right)}{\text{Rat}{e_{c=}}_{0}\left(t_{0}\right)}$$

where *Rate (t)* is the rate at time *t* before injection, *Rate*_*c* = 0_*(t)* is the control rate (concentration = 0), *t*_0_ is the last point before injection and *t’* is defined for each reagent; Gramicidin A, the time of maximum PPR for concentration of 20 μM, HMEC 40.8 minutes, MCF10a 40.8 minutes, MCF7 37 minutes, MDA-MB-231 27.2 minutes; Verapamil 116 minutes; Ouabain 20.4 minutes for both HMEC and MDA-MB-231 cells; and Mannitol 12 minutes.

### Engineered cell lines and PMA-1 construct

Yeast plasma membrane ATPase 1 (PMA1 [NM:001180873]) was cloned into pcDNA3.1 vector (it is named here pcDNA/PMA1). The details of the cloning will be shown in an upcoming paper. MCF7 cells as the transfection host cell line were acquired from American Type Culture Collection (ATCC HTB-22, Manassas, VA, 20108) and were cultured under standard cell culture conditions. The cells transfected with pcDNA or pcDNA/PMA1 vectors, respectively, resulted in MCF7/Mock and MCF7/PMA1 cell lines by a standard stable cell construction procedure.

### Western blotting

The cell membrane protein samples from the cultured MCF7/Mock and MCF7/PMA1 cells were collected using mem-per eukaryotic membrane protein extraction reagent kit (Thermo Scientific, MA, US 89826) according to the protocol instruction, and the protein samples were further purified and concentrated by pierce SDS-PAGE sample prep kit (Thermo scientific, 89888). Thirty micrograms of protein per sample was separated on polyacrylamide-SDS gels and electrophoretically transferred to nitrocellulose membranes. Membranes were incubated with primary antibody against PMA1 (1:1,000, Abcam, ab4645), and glyceraldehyde-3-phosphate dehydrogenase (GAPDH) (1:1,000, Santa Cruz Biotechnology, TX, US se-25778). For visualization, horseradish peroxiase (HRP)-conjugated secondary antibodies: Goat anti-rabbit IgG HRP and goat anti-mouse IgG HRP, followed by the ECL kit (Thermo Scientific, 32209) were used.

### Oxygen consumption and proton production rate measurements

OCR and PPR for MCF7, MCF7/Mock and MCF7/PMA1/Clone 1 cells were measured by using the Seahorse Extracellular Flux (XF-96) analyzer (Seahorse Bioscience). The cells were cultured for one hour in the absence of glucose in a non-CO2 incubator prior to the measurements. Then the PPR and OCR were measured in the absence of glucose associated with non-glycolytic activity, following three sequential injections of D-glucose (5 mM), oligomycin (1 μM), and 2-deoxyglucose (50 mM) in real time. Glycolytic PPR was determined by the difference between the PPR in the presence of glucose and in the absence of glucose. Protein concentration was determined for each well using a standard BCA protein assay. The OCR and PPR values are normalized to μg protein.

### Statistical analysis

**Figure 1:**
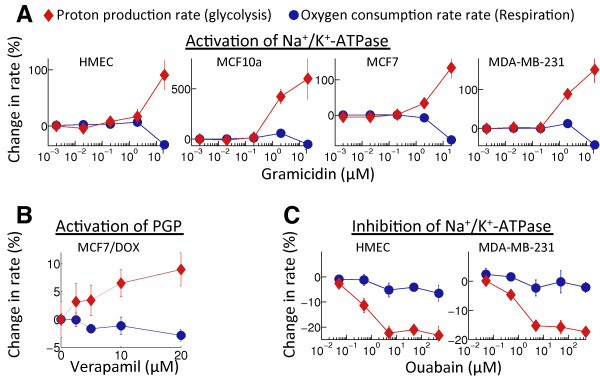
**Changes in proton production rates, indicating glycolytic activity, and oxygen consumption rates, corresponding to oxidative phosphorylation, following stimulation/inhibition of membrane transporters.** Changes in proton production rates are represented by red diamonds and oxygen consumption rates by blue circles. **(A)** Stimulation of Na^+^/K^+^-ATPase through increasing concentration of Na^+^/K^+^-ionophore Gramacidin A in normal human mammary epithelial cells (HMEC), and in dysplastic (MCF10A), non-metastatic (MCF7) and invasive, malignant (MDA-mb-231) cells results in increasing glycolytic activity and little change in oxidative phosphorylation except for a decline at the highest dose. **(B)** Stimulation of P-glycoprotein (PGP) pumps on the membrane of PGP-expressing MCF7 cells by Verapamil also results in increase of glycolytic activity. **(C)** Inhibition of membrane activity by increasing concentrations of Ouabain in both malignant (MDA-mb-231) and normal (HMEC) cells results in a decrease in glycolytic rates but no significant change in oxidative metabolism. Error bars represent SD (n = 6).

**Figure 2:**
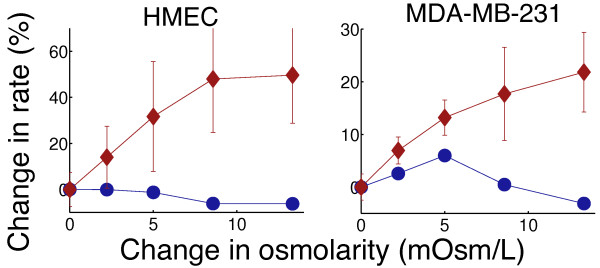
**Metabolic profiling of osmotic stress.** Relative changes in proton production rates (red diamonds) indicating glycolytic activity, and oxygen consumption rates (blue circles) corresponding to oxidative phosphorylation, following an increase in extracellular osmotic pressure using mannitol. For both human mammary epithelial cells (HMEC) and MDA-MB-231, glycolysis is the main energetic source for osmotic balancing across the plasma membrane. Error bars represent SD (n = 12).

**Figure 3:**
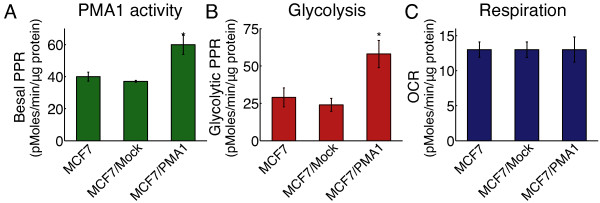
**Metabolic profile of adaptation to chronic increase of membrane transporter activity using plasma membrane ATPase 1 (PMA1)-cloned MCF7.** The proton production rate (PPR) and oxygen consumption rate (OCR) were measured in the indicated cell lines. **(A)** Basal, non-glycolytic, PPR, indicating H^+^ pump activity is significantly higher in the PMA1 clone compared to control cells MCF7 or mock (measured in the absence of glucose). **(B)** Glycolytic PPR was significantly higher in the PMA1 clone (2.1-fold, compared to control cells MCF7 or mock-transfected cells) but **(C)** there was no significant change in OCR. The data represent the mean ± standard error from three individual experiments. The two-tailed Student *t*-test was used to calculate statistical significance: *P <0.05; error bars represent SD (n = 4).

For metabolic profiling of membrane activity (Figures 1 and 2), each reagent concentration was measured six times; the error bars demonstrate SD. For metabolic profiling of PMA1-transfected cells (Figure 3), every measurement was repeated four times; the error bars demonstrate SD. The two-tailed Student *t*-test was used to calculate statistical significance.

### Computational modeling

For detailed description on computational modeling, see Additional file 1.

## Results

To investigate the separation of energy demand and production, we examined changes in glucose metabolism following perturbations in membrane transport activity under normoxic conditions in several cell lines, which represent the spectrum from normal breast epithelium to aggressive, metastatic cancer. We measured changes in OCR and glucose-dependent PPR, which indicate changes in rates of oxidative and glycolytic metabolism respectively. As demonstrated in Figure 1, the Na^+^/K^+^-ionophore Gramicidin A was added to culture media to increase Na^+^/K^+^-ATPase activity by disrupting the ionic balance. This led to significant increases in the glucose-dependent proton production rate, indicating upregulated glycolysis, while oxygen consumption rates were reduced or unchanged (Figure 1A). Similar results were obtained with the activation of P-glycoprotein (PGP) transporters, which pump lipophilic cationic xenobiotics out of cells, in PGP over-expressing MCF7 cells following addition of a substrate (Verapamil) to the culture medium (Figure 1B). In contrast, Ouabain was used to inhibit Na^+^/K^+^-ATPase in normal breast epithelial (HMEC) and breast cancer (MDA-MB-231) cells, and this led to significant decreases in glucose-dependent acid production while the oxygen consumption rates were not significantly affected (Figure 1C).

We also measured changes in glucose metabolism in response to acute osmotic stress, which increases the activity of membrane to regain the cell volume after the osmotic shrinkage [23-25]. Addition of mannitol to the media, both in normal breast epithelial (HMEC) and breast cancer (MDA-MB-231) cells led to an increase in glycolytic, compared to oxidative, metabolism (Figure 2). It is similarly notable that manipulations of macromolecule synthesis processes have been reported to affect respiration but not glycolysis [26].

**Figure 4:**
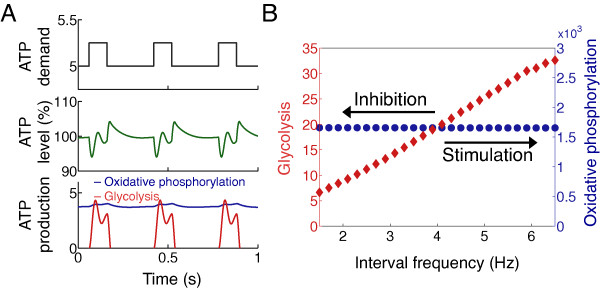
**Simulations of periodic intervals of an increased demand for ATP, at the region within 1 μm from the cell membrane, on a 1D cell (Additional file**1**: Figure S2). (A)** Top: periodic ATP demand; middle: ATP level (green line); bottom: mitochondrial ATP production (blue) and glycolytic ATP production (red). **(B)** Total ATP production rate of the glycolytic pathway (red triangles) and mitochondrial respiration rate (blue circles) at different pulse frequencies, where frequency increment/reduction represent activation/inhibition of membrane demand. While glycolytic production rate is highly dependent on the demand frequency, mitochondrial production rate remains relatively constant, similar to the experimental results shown in Figure 2.

These results were consistent with computational simulations of changes in short-timescale demand for energy by cell membrane processes (Figure 4A). In these simulations (supplemental Algorithm 1 in Additional file 1), we imposed periodic and acute energy demands (black bar), which we allowed to be satisfied by slow/efficient (blue) and rapid/inefficient (red) ATP generating processes. As shown here, there were large and significant increases in the rapidly responding (glycolytic) mechanism, with only slight changes in the slow (mitochondrial) mechanism (Figure 4B).

In addition, we examined the metabolic consequences of adaptation to chronic increase of membrane transporter activity without external perturbation using cloned MCF7 cells transfected with yeast plasma membrane ATPase, (PMA-1) [27]. Under normoxic conditions, PMA-1-transfected cells exported protons at approximately 150% the rate of mock transfectants or parental cells (Figure 3A), with the majority of energy for this process being supplied by greater than 2-fold increase in glucose-dependent proton production (Figure 3B), which is in contrast to the absence of differences in respiration rates (Figure 3C). This suggests that the increased reliance on glycolysis appears to be associated with energy demand at the membrane, even under chronic conditions. Nonetheless, we contend that this association has been evolutionarily selected because of the rapid response time afforded by glucose fermentation.

**Figure 5:**
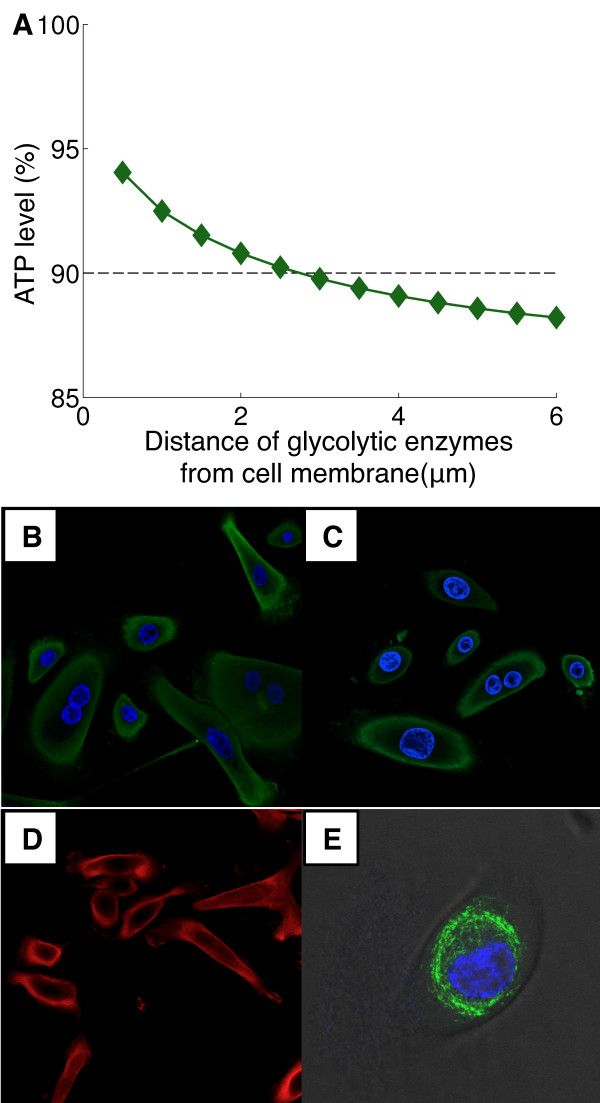
**Compartmentation of metabolic pathways in different sites in the cell. (A)** Simulation results demonstrating the decline of ATP due to a step-wise increase in demand at the cell membrane depending on the localization of the ATP-producing enzymes in the glycolytic pathway. To maintain the ATP concentration above the 90% threshold observed experimentally, the simulations demonstrate the source of ATP production must be concentrated next to the cell membrane. Immunocytochemical images of **(B)** pyruvate kinase, **(C)** phosphoglycerate kinase, **(D)** glyceraldehyde 3-phosphate dehydrogenase and **(E)** mitochondria in HMEC cells, demonstrating distinctive peripheral and distinct perinuclear distribution of the pathways respectively (blue, nuclei staining).

The different timescales of ATP-demand influence the spatial localization of the metabolic pathways. That is, ATP-producing enzymes in the glycolytic pathway must be localized near the plasma membrane (PM) to permit rapid supply of ATP directly to membrane pumps. Numerical simulations of a sudden increase in energy demand at the PM demonstrate that maintaining ATP concentration above 90% [28] requires localization of the rapid ATP-producing processes in the vicinity of the PM (Figure 5A). The numerical prediction that the two metabolic pathways are spatially separated is consistent with immunocytochemical imaging of the glycolytic enzyme, pyruvate kinase (Figure 5B), glyceraldehyde 3-phosphate dehydrogenase (Figure 5C) and phosphoglycerate kinase (Figure 5D), in HMEC cells indicate that their spatial distribution is in the periphery of the cytoplasm, adjacent to the PM. In contrast, mitochondria in HMEC cells are perinuclear (Figure 5E).

## Discussion

Our results demonstrate that glycolysis serves a critical physiological function in normoxic conditions by responding to rapid energetic demands that occur primarily in the cell membrane. We showed that inhibition or activation of transporters on the cell membrane led to reduction or increase in glycolytic activity, respectively, while the respiration rate remained unchanged. Furthermore, increased aerobic glycolysis is also observed in chronically increased membrane demand in cells with constitutively upregulated membrane pumps. Interestingly, this suggests that while increased acid export into the environment may be a consequence of upregulation of aerobic glycolysis, it may also be a cause.

In all of our experiments, we did not observe significant changes in OCR following membrane transporters manipulations. Therefore, regardless of additional processes that might influence the PPR measurements, manipulations of the energetic demands of the membrane transporters led to changes in glycolytic metabolism. Consistent with model prediction we showed that the spatial distribution of ATP-producing components of glycolysis (pyruvate kinase), rather than well-mixed in the cytoplasm, are primarily localized adjacent to the cell membrane, while mitochondria, responsible for aerobic metabolism, are typically perinuclear in mammalian cells [29-31]. Prior studies have similarly localized glycolytic metabolism adjacent to the membrane in muscle cells [32] and erythrocytes [33] and it has been suggested that this co-localization of the glycolytic enzymes expedites the production of ATP by metabolic channeling [34,35]. Interestingly, while the biochemical details of cellular glucose metabolism have been thoroughly investigated for decades [36-38], the spatial constraints on these complex dynamics are rarely considered [39].

**Figure 6:**
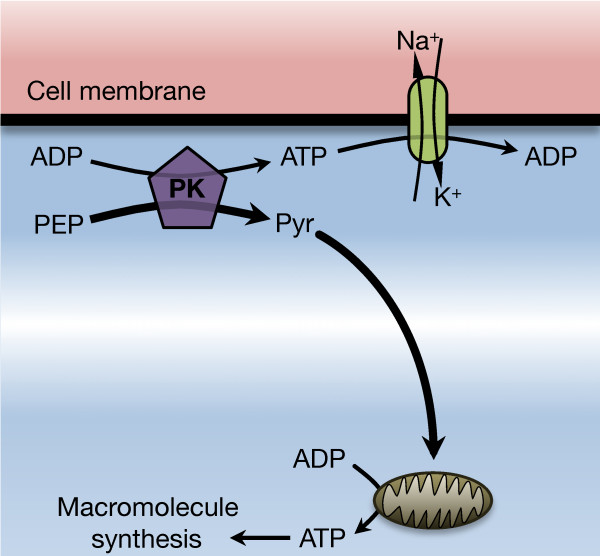
**Proposed metabolic model.** Glycolysis supplies energy for membrane components such as ion transporters and oxidative metabolism primarily supplies energy for macromolecule synthesis. (PER – Phosphoenolpyruvate; PYR – Pyruvate).

**Figure 7:**
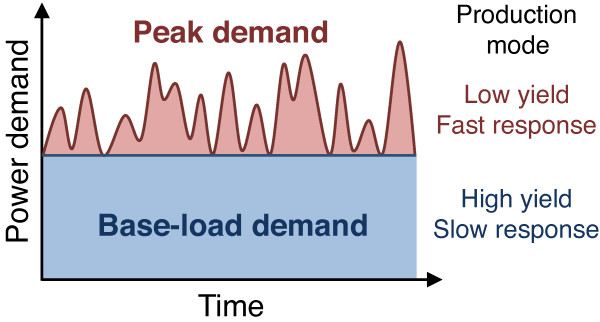
**Load curve diagram showing power demand over a course of time.** In optimal design of power grids, power demand is typically divided into base-load demand (blue), which is the continuous basal demand of power, and peak-load (red), short periods of an increased demand for energy. Different power plants with different efficiency and response time supply the two types of demands.

These results support an alternative, demand-based model of glucose metabolism, in which oxidative and fermentative metabolic pathways are complementary production modes that supply ATP for slowly responding basal demand and rapidly responding peak demand, respectively (Figure 6). Power grids provide a useful analogy. Economic analyses of strategies to minimize the total cost of power generation while satisfying variable real-time demand have demonstrated that maximal efficiency is achieved by dividing power demand into base-load and peak-load components [40] (Figure 7). Base load is the continuous energy demand of the system. Peak loads are the fluctuating component in energy demand superimposed on the base-load demand. The former is satisfied by coal or nuclear plants, which are efficient but slow to respond, whereas the latter is typically met by less efficient but fast-responding gas turbines. Thus, measurement of nuclear or coal power generation reflects the base demand of an electric grid and the activity of gas turbines is determined by the magnitude of fluctuating peak demand.

Similar to power grid economics, cells maintain the lowest possible baseline ATP production rates but with reserve production capability sufficient for rapid response to sustain critical adaptive functions. In our model of cell metabolism, oxidative phosphorylation supplies stable or slowly fluctuating demands with highly efficient conversion of carbons to CO_2_ and ATP. The penalty for high efficiency, however, is slow response to transient increases in demand. Mitochondrial respiration requires about 12 to 13 seconds [41-43] to respond to sudden changes in cell function or external perturbations, which, though fast, may be insufficient for critical processes. In contrast, the glycolytic pathway, although less efficient, can accelerate ATP production within milliseconds to provide ATP following high-amplitude or high-frequency increases in demand [44]. We do not dispute the importance of the Pasteur effect. Of necessity cells must switch to glycolysis in hypoxic conditions, but this does not mean that glycolysis serves no role in aerobic conditions. Returning to our power grid analogy, gas turbines supply energy for fluctuating energy and serve as back-up should base-load production fail. Similarly, in normal physiological conditions, glycolytic metabolism is maintained and its magnitude is governed by fluctuations in the energy demand primarily for membrane activity.

High frequency fluctuations in energy demand occur most commonly at the cell membrane, primarily for maintaining local osmotic balance across the cell membrane by energy-dependent water and ion fluxes. Failure of effective and rapid response to local changes in the osmotic balance across the cell membrane may result in unregulated membrane protrusion or impairment of membrane integrity; potentially causing lethal cell damage. Thus, the fast response of the glycolytic pathway serves a critical role, by responding to rapid fluctuations in energy demand [45-48] when oxidative phosphorylation may be insufficient to maintain the adequate levels of ATP necessary to permit cell survival following environmental fluctuation, or support rapid and diverse membrane changes required for cell movement.

In our model of glucose metabolism, glycolysis will be increased by any cellular processes that activate membrane pump activity, including changes in cell shape such as mitotic rounding [18], invasion through confined spaces [19], and motility [20,21]. Consistent with this, aerobic glycolysis is observed in numerous physiological processes that are associated with acute membrane events such as lymphocyte proliferation and antibody secretion [3,7], macrophages migration [8], embryonic cytokinesis and cell migration [4].

Furthermore, increased aerobic glycolysis in cancer cells may originate simply as a physiological response to increased fluctuations in energy demand due to enhanced membrane transporter activity [49], which is necessary for virtually every critical malignant phenotypic property [10] including proliferation, growth, migration and invasion, and requires increased membrane activity. Interestingly, Racker observed increased glycolysis following activation of the Na^+^/K^+^-ATPase [50] over 40 years ago and proposed that the Warburg effect was caused by a defect in the Na^+^/K^+^-ATPase, which caused it to operate inefficiently. However, this mechanism was unsubstantiated [51,52]. Our model, in contrast, proposes that aerobic glycolysis is not a disorder of metabolism but simply the physiological response to fluctuating energy demands within the membrane due to normal but upregulated activity of transporters required for cell division, growth, and migration.

We recognize that, regardless of the mechanism of its origin, increased aerobic glycolysis may lead to a broader dysregulation of cellular energetics during cancer evolution because it confers an additional selective advantage [53]. For example, increased glycolytic metabolism leads to increased production of hydrogen ions, which in combination with insufficient blood perfusion induce an acidic extracellular environment, which may promote invasion, and further the membrane energy demand in a vicious cycle [53-55]. In addition, It has been known since the early 20^th^ century [56] that pyruvate can be an efficient scavenger of reactive oxygen species [57,58] and thus, the pyruvate generated by glycolytic metabolism will protect cells from oxidative stress, especially prior to the S phase [59,60].

## Conclusion

In conclusion, we introduce a model wherein energy metabolism is primarily governed by the temporal dynamics of energy demand, and not by the availability of oxygen. We do not dispute the importance of the Pasteur effect, glycolysis is a necessary emergency back-up that permits cells to adapt to periods of hypoxia. We propose, however, that the converse is not true - cells use glycolysis even in the presence of oxygen to respond to fluctuating energy demands primarily in the membrane. In our model, the Warburg effect in cancer cells originates not as a disorder of cellular energetics but as a physiological response to meet energy from membrane activity in mobile, proliferative cells particularly in a temporally and spatially variable environment.

## Abbreviations

BSA: bovine serum albumin; DMEM: Dulbecco's modified Eagle's medium: FBS: fetal bovine serum; HMEC: human mammary epithelial cells; HRP: horseradish peroxiase; OCR: oxygen consumption rate; PBS: phosphate-buffered saline; PGP: P-glycoprotein; PM: plasma membrane; PMA-1: plasma membrane ATPase 1; PPR: proton production rate; PER: Phosphoenolpyruvate; PYR: Pyruvate.

## Competing interests

The authors declare that they have no competing interests.

## Authors’ contributions

TE conceived of the study, participated in its design, performed metabolic analysis and imaging, carried out the numerical studies and drafted the manuscript. LX transfected cells and carried out their metabolic analysis and helped to draft the manuscript. RJG conceived of the study, participated in its design and helped to draft the manuscript. RAG conceived of the study, participated in its design and helped to draft the manuscript. All authors read and approved the final manuscript.

## Supplementary Material

Additional file 1 **Computational modeling.** Description of data: description of numerical model including model equations, detailed simulation results and a Matlab code. Click here for file
