# Supplementary material for: Separation of metabolic supply and demand: aerobic glycolysis as a normal physiological response to fluctuating energetic demands in the membrane
Source: Cancer Metab. 2014 Jun 5;2:7. doi: 10.1186/2049-3002-2-7 (PMC4060846; doi:10.1186/2049-3002-2-7)
Supplement: Additional file 1 — Computational modeling. Description of data: description of numerical model including model equations, detailed simulation results and a Matlab code. [file 2049-3002-2-7-S1.pdf]

**Computational Modeling**

Finite element simulations were conducted on COMSOL 4.3. MATLAB 2012a was used for simulation analysis and plotting.

*Temporal dynamics*

ATP and ADP concentrations, $C_{ATP}$ and $C_{ADP}$ respectively, are governed by the following equations:

(E1)
$$\frac{\partial C_{ATP}}{\partial t} = -k_{dmn}(t) + a \cdot P \cdot 4C_{ADP}$$
$$\frac{\partial C_{ADP}}{\partial t} = +k_{dmn}(t) - a \cdot P \cdot 4C_{ADP}$$

$k_{dmn}(t)$ is the demand term, converting ATP to ADP, is the step function:

(E2)
$$k_{dmn}(t) = \begin{cases} d & t < 0.1\sec \\ d(1+F) & t \geq 0.1\sec \end{cases}$$

where $d$ is the basal demand and $F$ is the fluctuation magnitude.

The production term, $a \cdot P \cdot 4C_{ADP}$, converts ADP to ATP. It consists of 3 terms: production factor, $\alpha$, production rate, $P$, and the term, $4C_{ADP}$, which restricts the production of ATP at low levels of ADP.

The production rate, $P$, depends on the ratio between ATP and ADP concentrations:

(E3)
$$\frac{\partial P}{\partial t} = k\left(1 - \frac{C_{ATP}}{C_{ADP} \cdot S}\right)$$

$S$ is the ratio coefficients in which at that ratio the production rate remains constant. The metabolic response time, which depends on the rate coefficient, $k$, governs the transition time in response to changes in the ratio $C_{ATP}/C_{ADP}$.

The initial conditions were $C_{ATP}(0) = 2/3$, $A_{ADP}(0) = 1/3$, $S = 2$ with basal demand of $d = 5$ and production factor $\alpha = 1$. For these values, the production rate steady state is $P = 3.5$.

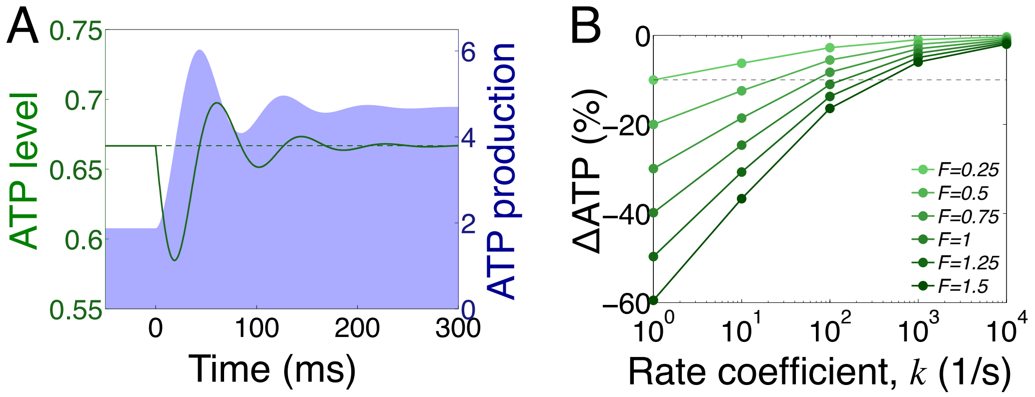


**Figure S1:** Temporal variations of ATP supply and production due to a rapid increase in energy demand. (a) Changes in ATP concentration (green) and ATP production (blue area) following an instantaneous increase of demand for ATP by factor of F at time t=0. The increased demand for ATP depletes the ATP level from its basal level (⅔), which in response increases the production of ATP production. (b) The magnitude of ATP initial depletion, ΔATP, depends on the magnitude of the increased demand, *F*, and the Rate coefficient, *k*. For temporal response of $R \geq 10^3$ the ATP depletion is less than 10% regardless of demand.

_Spatiotamporal simulation_

Figure S2 depicts the model system of a half 1D cell between the nuclear membrane and the cell membrane, with distance of 11μm. Mitochondrial production takes place throughout the cytoplasm uniformly and glycolytic production is confined to a glycolytic range next to the cell membrane. Cytosolic demand is the uniform basal demand across the cell and membrane demand occurs in a narrow region of 0.3μm next to the cell membrane.

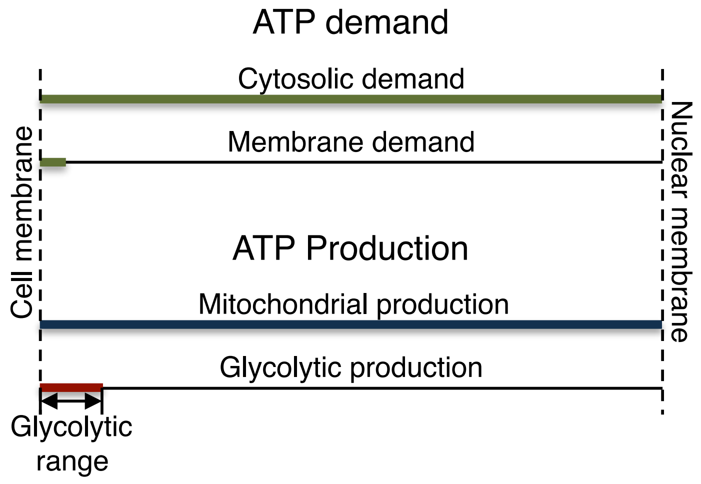


**Figure S2:** Schematic diagram of demand and production regions in 1D cell model system. Green represents demand regions, blue and red represent mitochondrial and glycolytic ATP production respectively. Glycolytic range is the region of glycolytic production.

The governing reaction-diffusion equations are similar to the temporal ones (Equations (E1) and (E3)) with no-flux boundary conditions:

(E4)
$$\frac{\partial C_{ATP}}{\partial t} = D\nabla^2 C_{ATP} - k_{dmn}(t) + \left(MP_m + GP_g\right)4C_{ADP}$$
$$\frac{\partial C_{ADP}}{\partial t} = D\nabla^2 C_{ADP} + k_{dmn}(t) - \left(MP_m + GP_g\right)4C_{ADP}$$
$$\frac{\partial P_{m/g}}{\partial t} = k_{m/g}\left(1 - \frac{C_{ATP}}{C_{ADP}\cdot S_{m/g}}\right)$$

The parameters are similar to the parameters of the temporal simulations, $C_{ATP}(0) = 2/3$, $A_{ADP}(0) = 1/3$, $d = 5$, $k_m = 50$, $k_g = 100k_m = 5000$, $S_m = 2$ and the glycolytic set-point was set to be 2% below the mitochondrial set-point, making the ratio coefficients to be $S_g = 1.8846$, so when there are no fluctuations in demand the system will move only to mitochondrial production. The mitochondrial production coefficient was $M = 1$ and the glycolytic production coefficient depends on the glycolytic range, $R_g$, such that $G = 0.3 / R_g$, where unless other stated, $R_g = 1\mu m$.

The glycolytic production rate, $P_g$ , was limited at from blow at $P_g = 0$, to avoid negative value, and with a maximum capacity of $P_g = G_{\max}$.

The diffusion coefficient was arbitrarily set to $D = 2\,\mu m^2/s$. This value is 2 orders of magnitude less the measured cytosolic nucleotide diffusion coefficient (Kinsey and Moerland, 2002) and 2 orders of magnitude higher than the ATP diffusion coefficient near the inner surface of the plasma membrane (Kabakov, 1998).

In the calculations of production rates vs. demand intervals (Figure 4), the simulation was running for 20 second and rate values were calculated by averaging the last 6 periods. Figure S2 shows the average production rates over the entire simulations. Above interval frequency of f=6.67, the demand on the membrane is more than 2 time longer in the increased state than in the basal demand, and as seen in Figure S3, above this frequency the portion of the mitochondrial production increases and the portion of the glycolytic production decreases until it reaches 0 in frequency f=10Hz which is a constant increased demand.

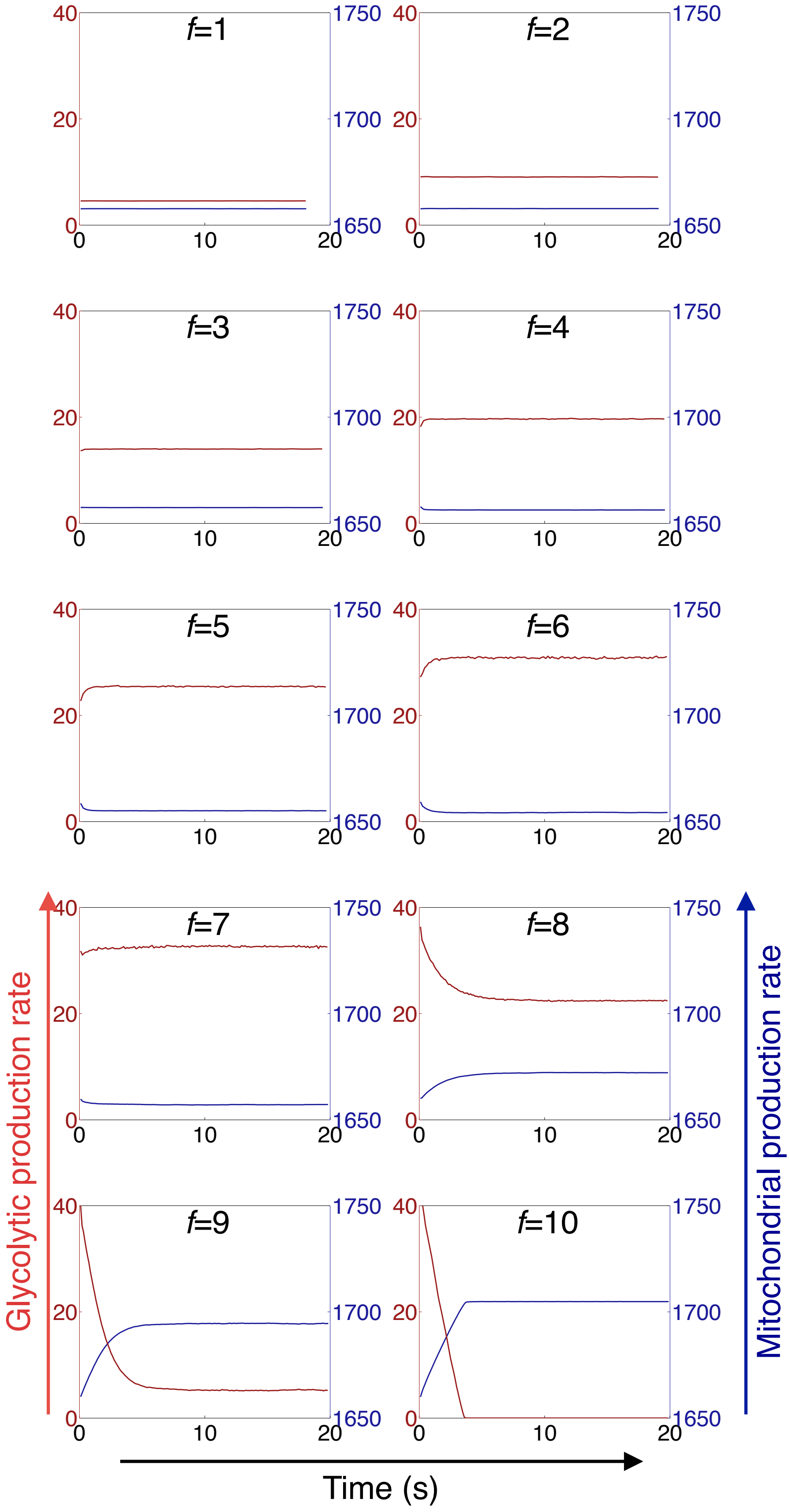


**Figure S3:** System stabilization at different interval frequencies. Glycolytic production rate (red) and mitochondrial production rate (blue) over an interval period.

The calculations of ATP depletion as function of the glycolytic range are highly dependent on the glycolytic maximum capacity, $G_{\max}$, and the fluctuation magnitude, $F$, as shown in Figure S4.

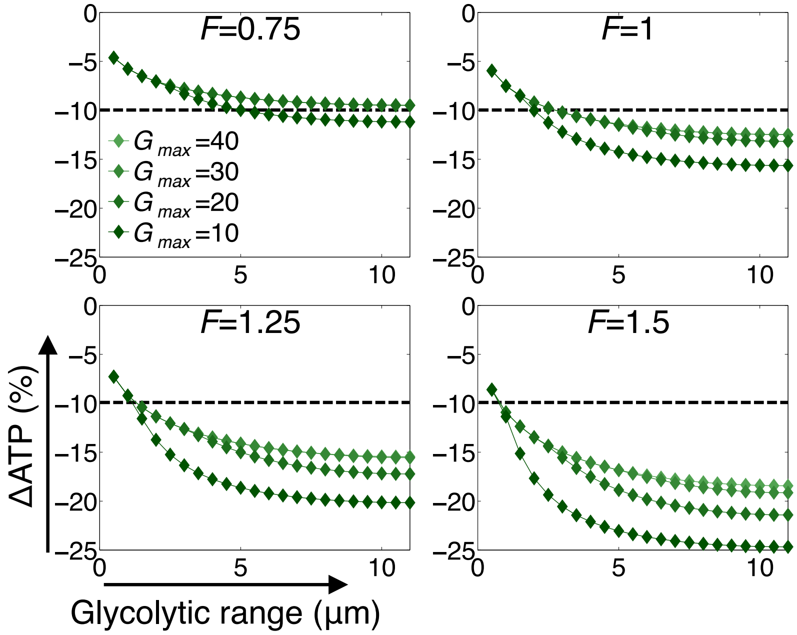


**Figure S4**: ATP depletion, $\Delta ATP$, versus glycolytic range at different fluctuation magnitude, $F$, and glycolytic capacity, $G_{\max}$.

## References


Kabakov, A. Y. (1998). Activation of KATP channels by Na/K pump in isolated cardiac myocytes and giant membrane patches. Biophys J *75*, 2858-2867.

Kinsey, S. T., and Moerland, T. S. (2002). Metabolite diffusion in giant muscle fibers of the spiny lobster Panulirus argus. J Exp Biol *205*, 3377-3386.

## Algorithm

```
function
Metabolic_Simulation_example(Mito,Glyco,Fluct,Rm,Rg,k,l,Cutoff,Spread,L_seg,L_st
ep)

disp([datestr(now),' - n=0'])

import com.comsol.model.*
import com.comsol.model.util.*

model = ModelUtil.create('Model');

model.param.set('D', '2e-12');
model.param.set('Rm', Rm);
model.param.set('Rg', Rg);
model.param.set('Cyto_Dmn', '5');
model.param.set('set_point', '2/3');
model.param.set('S', 'set_point/(1-set_point)');
model.param.set('set_point_Glyco',2/3-4/300);
model.param.set('Sg', 'set_point_Glyco/(1-set_point)');
model.param.set('Mito', Mito);
model.param.set('Glyco', Glyco*(11^2-10^2)/(11^2-(11-Spread)^2));
model.param.set('Fluct', Fluct);
model.param.set('Cutoff', Cutoff);
model.param.set('L_seg',L_seg);


model.modelNode.create('mod1');

model.func.create('step0_01', 'Step');
model.func('step0_01').model('mod1');
model.func('step0_01').set('location', '0.0005');
model.func('step0_01').set('smooth', '0.02');
model.func('step0_01').set('from', '0');
model.func('step0_01').set('to', '1');
model.func.create('step0_10', 'Step');
model.func('step0_10').model('mod1');
model.func('step0_10').set('location', '0.0005');
model.func('step0_10').set('smooth', '0.02');
model.func('step0_10').set('from', '1');
model.func('step0_10').set('to', '0');
model.func.create('stepG_10', 'Step');
model.func('stepG_10').model('mod1');
model.func('stepG_10').set('location', 'Cutoff');
model.func('stepG_10').set('smooth', '0.02');
model.func('stepG_10').set('from', '1');
model.func('stepG_10').set('to', '0');
model.func.create('stepG_01', 'Step');
model.func('stepG_01').model('mod1');
model.func('stepG_01').set('location', 'Cutoff');
model.func('stepG_01').set('smooth', '0.02');
model.func('stepG_01').set('from', '0');
model.func('stepG_01').set('to', '1');
model.func.create('rect1', 'Rectangle');
model.func('rect1').model('mod1');
model.func('rect1').set('lower', '0.0');
```

```
model.func('rect1').set('upper', '0.1');
model.func('rect1').set('smoothactive', false);

model.geom.create('geom1', 1);
model.geom('geom1').lengthUnit([native2unicode(hex2dec('00b5'), 'Cp1252') 'm']);
model.geom('geom1').feature.create('i1', 'Interval');
model.geom('geom1').feature.create('i2', 'Interval');
model.geom('geom1').feature.create('i3', 'Interval');
model.geom('geom1').feature('i1').set('p2', '10');
model.geom('geom1').feature('i2').set('p1', '10');
model.geom('geom1').feature('i2').set('p2', '10.7');
model.geom('geom1').feature('i3').set('p1', '10.7');
model.geom('geom1').feature('i3').set('p2', '11');
model.geom('geom1').run;

model.variable.create('var1');
model.variable('var1').model('mod1');
model.variable('var1').set('R0', '1[1/s]');
model.variable('var1').set('R', '5[1/s]');
model.variable('var1').set('T', '1[s]');
model.variable('var1').set('Dmn0', 'Cyto_Dmn*R0');
model.variable('var1').set('Delta', '1-(u1/(u2*S))');
model.variable('var1').set('Delta_g', '1-(u1/(u2*Sg))');

K=0.1:k/1000:1;
for x=1:length(K)
    cmd=['model.variable(',char(39),'var1',char(39),').set(',...
        char(39),'Dmn',num2str(x),char(39),', ',char(39),...
        'Fluct*Dmn0*rect1((t-',num2str(K(x)),')/T)',char(39),');'];
    eval(cmd)
end

cmd='Dmn1';
for x=2:length(K)
    str1=['+Dmn',num2str(x)];
    cmd=[cmd,str1];
end

cmd2=['model.variable(',char(39),'var1',char(39),').set(',...
    char(39),'Dmnx',char(39),', ',char(39),cmd,char(39),');'];
eval(cmd2)

model.physics.create('c', 'CoefficientFormPDE', 'geom1');
model.physics('c').field('dimensionless').component({'u1' 'u2'});
model.physics('c').feature.create('src1', 'SourceTerm', 1);
model.physics('c').feature('src1').selection.set([2 3]);
model.physics('c').feature.create('src2', 'SourceTerm', 1);
model.physics('c').feature('src2').selection.set([3]); %#ok<NBRAK>
model.physics.create('c2', 'CoefficientFormPDE', 'geom1');
model.physics.create('c3', 'CoefficientFormPDE', 'geom1');
model.physics('c3').selection.set([2 3]);



model.mesh.create('mesh1', 'geom1');
model.mesh('mesh1').feature.create('edg1', 'Edge');
model.mesh('mesh1').automatic(false);
model.mesh('mesh1').feature('size').set('hgrad', '1.05');
```

```
model.mesh('mesh1').feature('size').set('hcurve', '0.2');
model.mesh('mesh1').feature('size').set('table', 'cfd');
model.mesh('mesh1').feature('size').set('hmax', '0.055');
model.mesh('mesh1').feature('size').set('hmin', '2.2E-4');
model.mesh('mesh1').feature('size').set('hauto', '1');
model.mesh('mesh1').feature('size').set('hmax', '0.025');
model.mesh('mesh1').run;


model.physics('c').feature('cfeq1').set('c', {'D'; '0'; '0'; 'D'});
model.physics('c').feature('cfeq1').set('f', {'R0*(u3*Mito)*4*u2-Dmn0'; '-
R0*(u3*Mito)*4*u2+Dmn0'});
model.physics('c').feature('init1').set('u1', 'set_point');
model.physics('c').feature('init1').set('u2', '1-set_point');
model.physics('c').feature('src1').set('f', {'R0*(u4*Glyco)*4*u2'; '-
R0*(u4*Glyco)*4*u2'});
model.physics('c').feature('src2').set('f', {'-Dmnx'; 'Dmnx'});

model.physics('c2').feature('cfeq1').set('c', '0');
model.physics('c2').feature('cfeq1').set('f',
'Rm*R*Delta*(step0_01(u3)+(Delta>=0)*step0_01(-u3))');
model.physics('c2').feature('init1').set('u3', '3.75/Mito');

model.physics('c3').feature('cfeq1').set('c', '0');
model.physics('c3').feature('cfeq1').set('f',...
    'Rg*R*Delta_g*(step0_01(u4)*stepG_10(u4)+step0_01(-u4)*(Delta_g>=0))');



n=1;
strRANGE=['range(',num2str((n-
1)*L_seg),',',num2str(L_step),',',num2str(n*L_seg),')'];
model.study.create('std1');
model.study('std1').feature.create('time', 'Transient');
model.study('std1').feature('time').set('tlist',strRANGE );

model.sol.create('sol1');
model.sol('sol1').study('std1');
model.sol('sol1').attach('std1');
model.sol('sol1').feature.create('st1', 'StudyStep');
model.sol('sol1').feature.create('v1', 'Variables');
model.sol('sol1').feature.create('t1', 'Time');
model.sol('sol1').feature('t1').feature.create('fc1', 'FullyCoupled');
model.sol('sol1').feature('t1').feature.remove('fcDef');
model.sol('sol1').attach('std1');
model.sol('sol1').feature('st1').name('Compile Equations: Time Dependent');
model.sol('sol1').feature('st1').set('studystep', 'time');
model.sol('sol1').feature('v1').set('control', 'time');
model.sol('sol1').feature('t1').set('control', 'time');
model.sol('sol1').feature('t1').set('tlist', strRANGE);
model.sol('sol1').runAll;
pd1=mpheval(model,{'t','u1','u3','u4','Dmnx'}, 'dataset', 'dset1'); %#ok<NASGU>
save('std1.mat','pd1')
disp([datestr(now),' - n=',num2str(n)])

for n=2:ceil(l/L_seg);
    strSTD=['std',num2str(n)];
    strSTDprev=['std',num2str(n-1)];
```

```
    strSOL=['sol',num2str(n)];
    strSOLprev=['sol',num2str(n-1)];
    strRANGE=['range(',num2str((n-
1)*L_seg),',',num2str(L_step),',',num2str(n*L_seg),')'];
    strDATASET=['dset',num2str(n)];

    model.study.create(strSTD);
    model.study(strSTD).feature.create('time', 'Transient');
    model.study(strSTD).feature('time').activate('c', true);
    model.study(strSTD).feature('time').activate('c2', true);
    model.study(strSTD).feature('time').activate('c3', true);
    model.study(strSTD).feature('time').set('tlist', strRANGE);

    model.sol.create(strSOL);
    model.sol(strSOL).study(strSTD);
    model.sol(strSOL).attach(strSTD);
    model.sol(strSOL).feature.create('st1', 'StudyStep');
    model.sol(strSOL).feature.create('v1', 'Variables');
    model.sol(strSOL).feature.create('t1', 'Time');
    model.sol(strSOL).feature('t1').feature.create('fc1', 'FullyCoupled');
    model.sol(strSOL).feature('t1').feature.remove('fcDef');
    model.sol(strSOL).feature('st1').set('study', strSTD);
    model.sol(strSOL).feature('st1').name('Compile Equations: Time Dependent');
    model.sol(strSOL).feature('st1').set('studystep', 'time');
    model.sol(strSOL).feature('v1').set('control', 'time');
    model.sol(strSOL).feature('t1').set('control', 'time');
    model.sol(strSOL).feature('t1').set('tlist', strRANGE);
    model.sol(strSOL).feature('v1').set('initmethod', 'sol');
    model.sol(strSOL).feature('v1').set('initsol', strSOLprev);
    model.sol(strSOL).feature('v1').set('solnum', num2str(L_seg/L_step+1));

    model.sol(strSOL).runAll;

    pdtemp=mpheval(model,{'t','u1','u3','u4','Dmnx'}, 'dataset', strDATASET);
%#ok<NASGU>
    cmd=['pd',num2str(n),'=pdtemp;'];eval(cmd)
    sName=['std',num2str(n),'.mat']; %#ok<NASGU>
    cmd=['save(sName,',char(39),'pd',num2str(n),char(39),')'];eval(cmd)

    model.study.remove(strSTDprev);
    disp([datestr(now),' - n=',num2str(n)])

end
```
